# Density-dependent cooperative non-specific binding in solid-phase SELEX affinity selection

**DOI:** 10.1093/nar/gkt477

**Published:** 2013-05-21

**Authors:** Abdullah Ozer, Brian S. White, John T. Lis, David Shalloway

**Affiliations:** Department of Molecular Biology and Genetics, Cornell University, Biotechnology Building, Ithaca, NY 14853,USA

## Abstract

The non-specific binding of undesired ligands to a target is the primary factor limiting the enrichment of tight-binding ligands in affinity selection. Solution-phase non-specific affinity is determined by the free-energy of ligand binding to a single target. However, the solid-phase affinity might be higher if a ligand bound concurrently to multiple adjacent immobilized targets in a cooperative manner. Cooperativity could emerge in this case as a simple consequence of the relationship between the free energy of binding, localization entropy and the spatial distribution of the immobilized targets. We tested this hypothesis using a SELEX experimental design and found that non-specific RNA aptamer ligands can concurrently bind up to four bead-immobilized peptide targets, and that this can increase their effective binding affinity by two orders-of-magnitude. Binding curves were quantitatively explained by a new statistical mechanical model of density-dependent cooperative binding, which relates cooperative binding to both the target concentration and the target surface density on the immobilizing substrate. Target immobilization plays a key role in SELEX and other ligand enrichment methods, particularly in new multiplexed microfluidic purification devices, and these results have strong implications for optimizing their performance.

## INTRODUCTION

Ligand selection based on affinity to solid-phase (immobilized) targets is commonly used for biochemical selections. For example, the target might be a signaling molecule, and the ligand mixture might consist of a biological extract with the goal being the purification of a high-affinity receptor (1). Alternatively, the target might be a peptide with the ligand mixture consisting of a random library of RNA or DNA aptamers as in the SELEX procedure (2). In all cases, ligand-target binding involves both weak non-specific interactions that are common to most ligands and strong specific interactions that are possessed only by the desired high-affinity ligands. The difference in strength between the non-specific and specific interactions (along with other factors) governs performance and can be quantitatively analyzed to determine the optimal ligand and target concentrations (3–8).

We draw attention here to an effect resulting from target immobilization that could also be an important performance factor: immobilization might increase the surface density of target molecules to the point where one ligand could concurrently interact non-specifically with multiple targets. Concurrent specific binding to multiple targets is unlikely in most cases because the probability of having two high-affinity binding sites on one ligand and having two adjacent targets in the correct orientation for concurrent specific binding is small. However, multi-target non-specific binding could increase otherwise low affinity: once a ligand has been localized by binding to the first target, binding to nearby targets will require no further spatial entropy loss, and therefore, more bindings are likely to occur. Such subsequent density-dependent cooperative (DDC) binding could alter the relative binding of high- and low-affinity ligands and affect the level of purification. It is impossible to theoretically evaluate these possibilities—too many relevant factors are unknown. For example, the effective surface area of agarose beads, which are commonly used for target immobilization, is difficult even to estimate because of pores and convolutions, and we do not know the extent to which the motion of substrate-bound targets might affect concurrent binding to multiple ligands. Thus, the first step is to experimentally determine whether cooperative binding to multiple targets can occur at all in realistic conditions.

We have explored this possibility in the context of affinity selection, as it is used in SELEX, although the results may be translated to other applications. SELEX uses multiple rounds of selection to isolate from a random library those aptamers with the highest target-binding affinities. Each round involves aptamer binding to either soluble or immobilized targets and a method that separates target-bound from -unbound aptamers. Immobilized targets have included proteins (9,10), peptides (11,12) and metabolites (13–15), and immobilization plays an essential role in new microfluidic SELEX technologies (10,16). Optimization of SELEX in these situations requires that we understand whether cooperative binding might change effective affinities, and if so, how.

To address this, we measured the non-specific binding of individual aptamers or a random aptamer library to bead-immobilized peptide targets. Binding curves displayed clear evidence of cooperative binding and showed that concentrating the targets on bead surfaces can increase the effective binding affinity of (undesired) low-affinity aptamers by two orders-of-magnitude. To understand this phenomenon, we showed that it can be quantitatively explained by a statistical mechanical model of DDC binding of a variable number of targets by a ligand. This model should be useful for analyzing the effects of cooperative binding in solid-phase affinity experiments such as SELEX and for optimizing experimental design by minimizing unwanted cooperative enhancement of low-specificity interactions.

## MATERIALS AND METHODS

### Immobilized peptides and radiolabeled aptamers

The H3-C peptide sequence matched the amino-terminal sequence of histone H3 (UniProt ID: Q71DI3) with an added cysteine residue at the C-terminal end for coupling: ARTKQTARKSTGGKAPRKQLATKA-C. H3K4me3-C was the same except that it was trimethylated at lysine 4. The peptides, synthesized by GenScript, were attached to Activated Thiol-Sepharose 4B resin (T8512, Sigma) via disulfide bonds: Beads were swollen as described by the manufacturer and stored in 0.1 M Tris–HCl (pH 7.5), 0.5 M NaCl, 1 mM EDTA with 20% ethanol at 4°C. Beads were washed 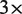 with 10 bead-volumes of 1X PBS, incubated with appropriate amounts of peptide (see later in the text) in PBS at room temperature with shaking for 8 h, and washed and stored in diethylpyrocarbonate-treated 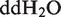 before the binding reactions. The amount of peptide linked to the beads was determined by measuring soluble peptide concentrations before and after the linkage reactions using the Qubit Protein Assay Kit (Invitrogen). Linkage efficiency was 75–90%. The highest target/bead ratio used, 21 μg/μl packed beads, was ∼80% of the published maximum binding capacity of the beads (17).

The N70 library was chemically synthesized by GenScript (Piscataway, NJ) with 26 and 24 nt constant regions flanking a 70 nt random region as described (18). NS1 and NS2 were selected from this library using the H3-C-unrelated targets Ublcp1 (Uniprot ID:Q8WVY7) and Chk2 (Uniprot ID:096017), respectively. Aptamers were radiolabeled to specific activities of 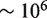 cpm/pmole by *in vitro* transcription using home-made T7 RNA polymerase and DNA templates containing T7 promoter (19).

### Binding titrations

Binding reactions were incubated for 3 h with continuous gentle inversion at 20°C with 2 nM 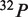-labeled aptamer and 30 μl of packed peptide beads in 0.5 ml SELEX buffer [10 mM HEPES–KOH (pH 7.6); 125 mM NaCl; 25 mM KCl; 1 mM 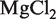; 0.2% Tween-20] prepared in diethylpyrocarbonate-treated water. (Pilot experiments demonstrated that binding equilibrium was achieved to 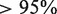 by 1 h.) Beads were then washed 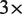 with 1 ml of SELEX buffer, and the amount of bound aptamer was measured by scintillation counting. For the constant target surface density (CTSD) experiments, a single batch of peptides linked at the highest target concentration was prepared, and aliquots were diluted with target-free (carrier) beads to obtain the specified total target concentrations using a total of 30 μl of packed beads. For the varying target surface density (VTSD) experiments, beads were linked to the appropriate amounts of peptide in separate reactions, and 30 µl aliquots were used in the binding reactions.

## RESULTS

### Binding at constant and varying target surface densities

**Figure 1.**
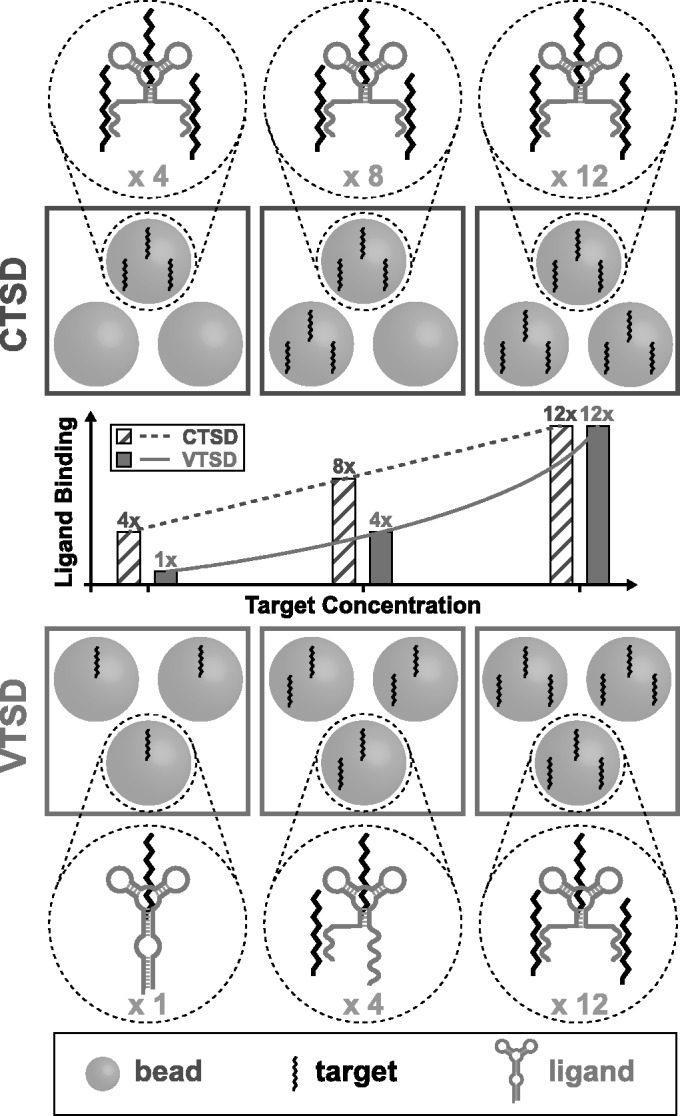
VTSD and CTSD experiments. As target concentration varies, the number of target-linked beads is kept constant in the VTSD experiment, whereas TSD is kept high and constant in the CTSD experiment by diluting target-linked beads with target-free carrier beads. The beads contain many randomly located targets at the figuratively illustrated densities. The large circles illustrate the binding by each ligand to an increasing number of targets as TSD increases. (Although the average number of targets bound by a ligand is illustrated, a ligand may bind any number of targets up to the maximum at every concentration.) The target concentration varies (left to right) from 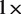 to 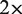 to 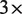; it is the same in the corresponding CTSD and VTSD panels, but the TSDs are different except at the highest concentration. At the lowest target concentration, the TSD is 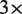 higher in the CTSD experiment than in the VTSD experiment, and this causes (with the binding-parameters used in this case) 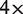 more ligands to be bound. In the CTSD experiment, the number of ligands bound increases linearly with target concentration (from 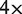 to 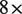 to 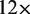) because the contribution from the cooperative effect does not change when TSD is held constant. In contrast, the increasing TSD in the VTSD experiment causes the number of ligands bound to increase more rapidly with target concentration (from 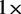 to 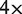 to 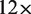) because the cooperative effect is also increasing.

To test for cooperative binding, we measured ligand retention in experiments with fixed aptamer concentration, varying (volumetric) target concentration, *T*, and either fixed or varying target-linked bead concentrations, *s* (Figure 1). In the first type of experiment, varying amounts of target were covalently linked to a fixed number of beads (i.e. corresponding to a fixed *s*) to obtain a series of decreasing target concentrations with varying target surface density (VTSD) and then incubated with aptamer ligands. The second type of experiment was a control in which a single batch of beads having target linked at the highest surface density used in the VTSD experiment was progressively diluted with target-free carrier beads to obtain the same series of decreasing target concentrations, but with a proportionate decrease in *s* that resulted in a constant target surface density (CTSD).

If there were no cooperative effects, the fraction of ligand bound would depend only on *T* and would be independent of the target surface density (TSD). In this case, the VTSD and CTSD binding curves would be the same and would be described by the law of mass-action (i.e. the Langmuir equation) (20),
(1)
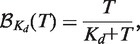

with the substitution 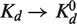, where 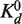 is the same ‘non-cooperative dissociation constant’ in both cases. The Langmuir binding curve has the familiar convex shape of the Michaelis–Menten function—it has slope 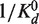 for small *T* and asymptotically saturates to one as *T* gets large. On the other hand, if binding were cooperative, we would expect VTSD binding to increase faster with *T* than is implied by Equation (1) because of the coordinately increasing TSD. This would change the shape of the binding curve and could, if cooperativity were strong enough, introduce positive curvature and an inflection point. In contrast, the control CTSD binding curve would still have the form of Equation (1), but with the substitution 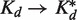, where 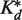 is a smaller ‘cooperative dissociation constant’ because of the constant enhancement of binding affinity resulting from the constant, high TSD. Because of this, the CTSD binding would be larger than the VTSD binding for every 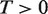, except at the highest value where the TSDs were the same.

We measured VTSD and CTSD binding using a random aptamer library and two individual RNA aptamers and immobilized peptide targets under conditions typical of those used in SELEX. The targets were a 25 amino acid peptide, H3-C, which was modeled on the amino terminus of histone H3 and a modified peptide, H3K4me3-C, which was trimethylated at an internal lysine. These were linked to agarose beads by disulfide linkage via the C-terminal cysteines that had been added for this purpose. In accord with the protocol described earlier in the text, the CTSD experiments used target-linked beads having a TSD of ≈(50 µM target)/(30 µl beads) that were diluted with target-free carrier beads to obtain the various volumetric target concentrations using a total (i.e. target-linked plus target-free) of 30 µl beads in the reaction volume. Conversely, in the VTSD experiments, varying amounts of target were bound to the entire 30 µl of beads; therefore, the TSD varied from ≈(50 µM target)/(30 µl beads) at the highest target concentration down to ≈(0.0024 µM target)/(30 µl beads) at the lowest non-zero target concentration.

The RNA aptamer library, N70, consisted of 120 nt aptamers having a central 70 nt random region flanked by constant regions on both ends. The individual aptamers, NS1 and NS2, had previously been selected from the N70 library using targets that were unrelated to H3-C and therefore, like the library, were presumed to bind non-specifically. After target-linked beads had been incubated with 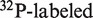 aptamers for 3 h (which exceeded the time required for equilibrium binding), they were washed, and the amount of retained aptamer was measured by scintillation counting. The experiments were conducted in great target excess; therefore, there was no significant difference between total and unbound target concentrations.

**Figure 2.**
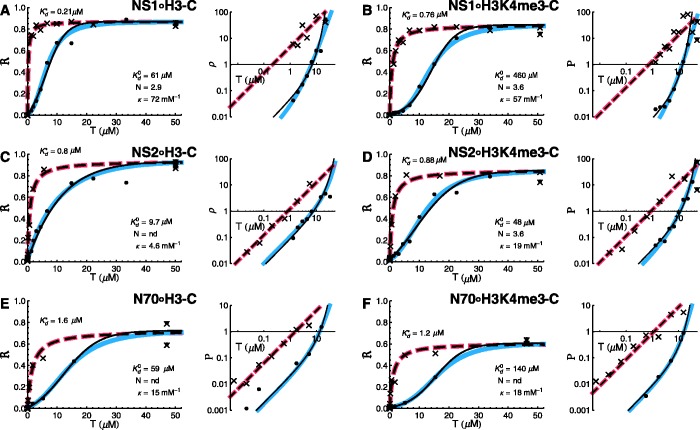
CTSD and VTSD retention of aptamers NS1 and NS2 and random aptamer library N70 to immobilized peptides. The left and right subpanels show, for the indicated aptamer-peptide pairs, plots of the retention functions 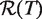 and log–log plots of the bound/unbound ligand ratio 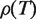, respectively. ‘CTSD experiments’ (×): The dashed thin black and wide red lines are the weighted least-square fits (see Supplementary Data section A obtained using either the Langmuir or complete DDC models, respectively. (The lines are superimposed in all cases.) ‘VTSD experiments’ (black circle): The thin black and wide blue lines are the weighted non-linear least-square fits using either the asymptotic or complete DDC models, respectively. Both the red and blue lines are determined by a single set of DDC parameters. The best-fit parameters (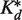 for the Langmuir model and 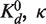, and *N* for the complete DDC model) are displayed (see Supplementary Table S1 for standard errors). All the data points were included in the nonlinear regressions, but not all are included in the log–log plots because the transform that defines 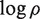 is hypersensitive to experimental fluctuations near 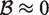 and 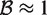. The κ units assume that *T* is expressed as a molarity and *s* as the fraction of the reaction volume occupied by the packed beads. nd: not determined accurately—in these cases, the standard error is greater than half the estimated value.

The results are shown in the left subpanels of Figure 2, which plot 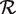, the fraction of aptamer retained after binding and washing, as a function of *T*. 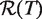 differs from 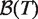 because some target-bound aptamers are lost during the wash and because background from aptamers that bind to the apparatus is also included. However, as quantified later in the text, 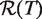 and 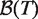 are linearly related; therefore, we can qualitatively compare the retention fractions in the same way as binding fractions.

Because the target-immobilizing surface area, and hence TSD, was the same at the highest target concentration tested in the VTSD and CTSD experiments (≈50 µM), binding at these points was the same within experimental error. However, the CTSD retentions (×) were notably higher than the VTSD retentions (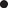) at all lower (non-zero) target concentrations, indicating that their higher TSDs increased binding affinity. For example, compare the CTSD and VTSD NS1∘H3-C binding at *T* = 1.3 µM: as discussed earlier in the text, the CTSD TSD was ≈50 µM target/30 µl beads, which was 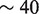 times higher than the VTSD TSD of 1.3 µM target/30 µl beads. This caused the CTSD aptamer retention to be 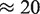 times higher than the VTSD retention. Moreover, it is evident that, with the exception of 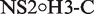, the shapes of the VTSD and CTSD curves are different: the CTSD curves have negative curvature everywhere, whereas the VTSD curves display some positive curvature and inflection points. As we show quantitatively later in the text, the CTSD curves can, as predicted, be well-fit by the Langmuir equation, whereas, as is qualitatively evident from their altered shapes, all but one of the VTSD curves cannot. Moreover, even the exception to this rule, 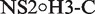 displays higher affinity in the CTSD experiment than in the VTSD experiment. In summary, the qualitative analysis showed that increasing TSD increases binding affinity, but we still needed a quantitative explanation of the mechanism responsible for this and for the variability in binding curve shapes.

### Theoretical modeling

The most likely explanation for the effect of TSD on binding affinity is, as explained earlier in the text, cooperative multi-target binding. To explore this hypothesis, we developed a quantitative model of DDC binding. The mathematical derivations are presented in Supplementary Data; here, we summarize the theoretical results and compare them with the data.

The total fraction of ligand that is retained after binding and washing can be modeled by
(2)
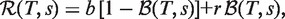

where *b* is the fraction of target-unbound ligand that is retained, *r* is the fraction of target-bound ligand that is retained, and binding and retention are now recognized to depend on both *T* and target-linked bead concentration, *s*. To facilitate graphical testing of the hypothesis that CTSD, but not VTSD, binding can be described by the Langmuir equation with the substitution 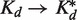, we define the ‘bound/unbound ligand ratio’
(3)
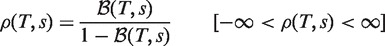

and rewrite the Langmuir equation (Equation 1) as
(4)
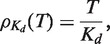

where the irrelevant argument *s* has been deleted. (Here and later in the text, we redefine ρ and 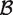 by context with arguments and subscripted parameter symbols as appropriate to the model being considered.) The log-log plot of 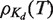 is a straight line with slope one, which makes it easy to analyze.

We used these equations to evaluate the ability of the Langmuir equation to fit the CTSD and VTSD data. Subjectively excellent fits to the CTSD data were obtained with 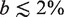 and 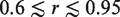 in all the experiments. (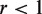 possibly because of aptamer loss during the washes; see Supplementary Data section B.) The fits to 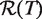 are displayed as dashed lines in the left subpanels in Figure 2; the corresponding log–log fits to 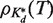 are shown in the right subpanels. The log–log fits all have slope one and cooperative dissociation constants, 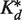 (corresponding to the 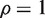 intercepts), that ranged from 0.21 to 1.6 μM.

On the other hand, it is evident that, except for 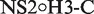, the log–log plots of the VTSD data cannot be well-fit by straight lines with slope one, i.e., by the Langmuir equation. Furthermore, when the 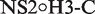 VTSD data are fit using the Langmuir equation, the estimated 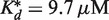 is 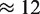 times larger than the CTSD 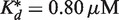. This shows that, even in this case, there is at least an order-of-magnitude increase in affinity because of the higher TSD.

### The DDC model of cooperative binding

Although the aforementioned analysis showed that the CTSD experiments were well-fit by the Langmuir equation, it did not explain the VTSD binding curves or the difference between the VTSD and CTSD affinities. To this end, we developed a biophysical model of cooperative non-specific solid-phase binding that explains the influence of TSD on binding affinity in a simple manner. We assume that a ligand can bind multiple colocalized targets sequentially and that the free energy of ligand binding to the first immobilized target includes a favorable enthalpic change but an unfavorable entropic contribution from localization of the ligand to the surface of the bead (and possibly other one-time processes such as aptamer unfolding or refolding):
(5)
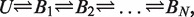

where *U* represents the unbound state and *B_n_* represents the state in which the ligand is bound to *n* targets. Once the ligand is bound to a target on the surface, its interactions with additional nearby targets do not require additional reductions in localization entropy; therefore, subsequent binding to them is even more favorable. To simplify the model, we assume that these 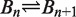 bindings all have the same ‘residual binding free-energy’, which is approximately the ‘intrinsic binding energy’ (21) of the aptamer in the structure (i.e. unfolded or folded) that is appropriate for non-specific binding (see Supplementary Data section C). The TSD determines the probability distribution of the number of colocalized targets and, therefore, in combination with the residual binding free-energies, determines the mean number of targets bound per ligand and hence, the effective affinity. This number will increase with increasing TSD up to a limit denoted by *N*, the maximum number of targets than can be bound by one ligand. (*N* will depend, at least in part, on the relative sizes of the ligand and target.)

### Asymptotic DDC model

The derivation of the binding equation corresponding to Equation (5) is simpler when the target is much smaller than the ligand (as in the experiments performed here, see ‘Discussion’ section). Then, we can view the targets as being randomly distributed on the bead surface like raindrops on the ground, and their spatial concentration can be described by the Poisson distribution (see Supplementary Data section C for details). In addition, if the TSD is modest, so that the mean number of binding partners is significantly less than *N*, we can simplify the analysis by ignoring *N*. Then, we get the simple ‘asymptotic DDC equation’ that depends on only two parameters—the non-cooperative dissociation constant 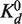, which is determined by the free-energy of the first binding, and the ‘asymptotic cooperativity constant’ 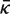, which depends on the free-energy of subsequent binding to additional targets.
(6)
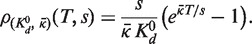



In a VTSD experiment, *s* is held at a constant value, which was s* = 60 µl packed beads/ml reaction volume in the experiments performed here. Therefore, the dependence of the bound/unbound ligand ratio on *T* is
(7)
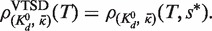

When *T* is small, 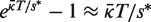 and 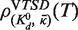 grows linearly with *T*:

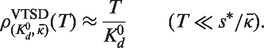

This has the same form as the Langmuir equation (Equation 4) with the substitution 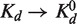 and occurs because ligands bind single targets non-cooperatively in this low-TSD situation. However, as *T* gets larger than 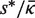, the exponential increase in 
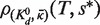
, which results from cooperative binding of a ligand to multiple targets, becomes noticeable. Accordingly, the log–log slope, 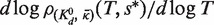, increases from one to 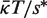.

This behavior is observed in the fits of the asymptotic DDC equation to the VTSD data, which are displayed as solid lines in Figure 2. Moreover, the model explains how effective binding affinity is increased in the 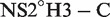 VTSD experiment without the appearance of an inflection point: As shown in Supplementary Data section C, binding will be increased whenever 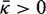, but there will be an inflection point only if 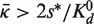. (The physical correlate is that the probability of binding two targets is greater than the probability of binding one target at 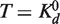 when 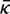 satisfies this inequality.) For 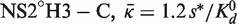; therefore, there is an increase in affinity, yet no inflection point. These results support the hypothesis that the effect of TSD results from DDC binding of an aptamer to multiple targets.

### Complete DDC model

We cannot apply the asymptotic DDC model to the CTSD data because it was collected in the high-TSD regime where the limitation on the number of bound targets can not be ignored. (The high-TSD inaccuracy is not evident in the asymptotic DDC model VTSD retention curves because binding is already saturated in this region.) To get accurate results in this regime, we developed the complete DDC model. It recognizes that *N*, the maximum number of targets that can be bound by one ligand, will be limited because of the finite sizes of the targets and ligand (and possibly other factors). As explained in Supplementary Data section D, the ‘DDC binding equation’ is
(8)
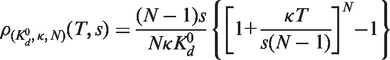

where κ is the ‘cooperativity constant’. This is close to asymptotic DDC binding as long as the mean number of targets bound is much less than *N*. (Equation 8 approaches Equation 6 as 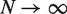, because 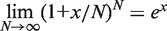.)

Analogous to Equation (7), Equation (8) with 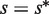 is used to model the VTSD data:
(9)
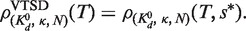

Because Equation (8) is also accurate at high TSDs, we can also use it to model the CTSD data. In this case, *s* is proportional to *T*. It equals 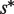, the fixed target-linked bead concentration used in the VTSD experiments, when *T* equals 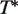, the highest target concentration used in both the VTSD and CTSD experiments; therefore, 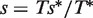. Substituting this into Equation (8) shows that the DDC CTSD equation is a linear function of *T* and can be rewritten in the Langmuir form
(10)
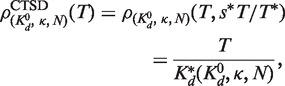

where 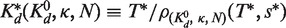.

To get the most accurate parameter estimates, we used Equations (9) and (10) together to simultaneously fit the VTSD and CTSD data for each aptamer–peptide pair using a single set of parameters. (For this purpose, we allowed *N* to be non-integer effective parameter. This corresponds to allowing a smooth rather than sharp cutoff in the maximum number of ligand-binding partners.) The best-fit curves are the wide dashed and solid colored lines in Figure 2. The CTSD fits are essentially identical to those obtained using the Langmuir model; in each case, the Langmuir 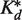 and the DDC 
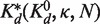
 are identical to each other within experimental error (Supplementary Table S1). The VTSD fits are similar to the asymptotic DDC ones except that, as expected, they do not rise as fast to the asymptote because the number of binding partners is limited.

The excellent quality of the fits supports the hypothesis that the observed cooperative binding can be explained by the DDC mechanism and gives confidence to the estimated parameter values. In particular, using the complete DDC equation allowed us to estimate the maximum number of ligand-binding partners, which is of biophysical interest. The estimated values of *N* were 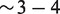 in all the experiments, indicating that a significant number of targets were cooperatively bound. In addition, the non-cooperative 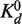
’s were from one to two orders-of-magnitude higher than the cooperative 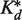
’s. The largest enhancement, observed for 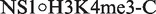, was 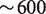-fold.

**Figure 3.**
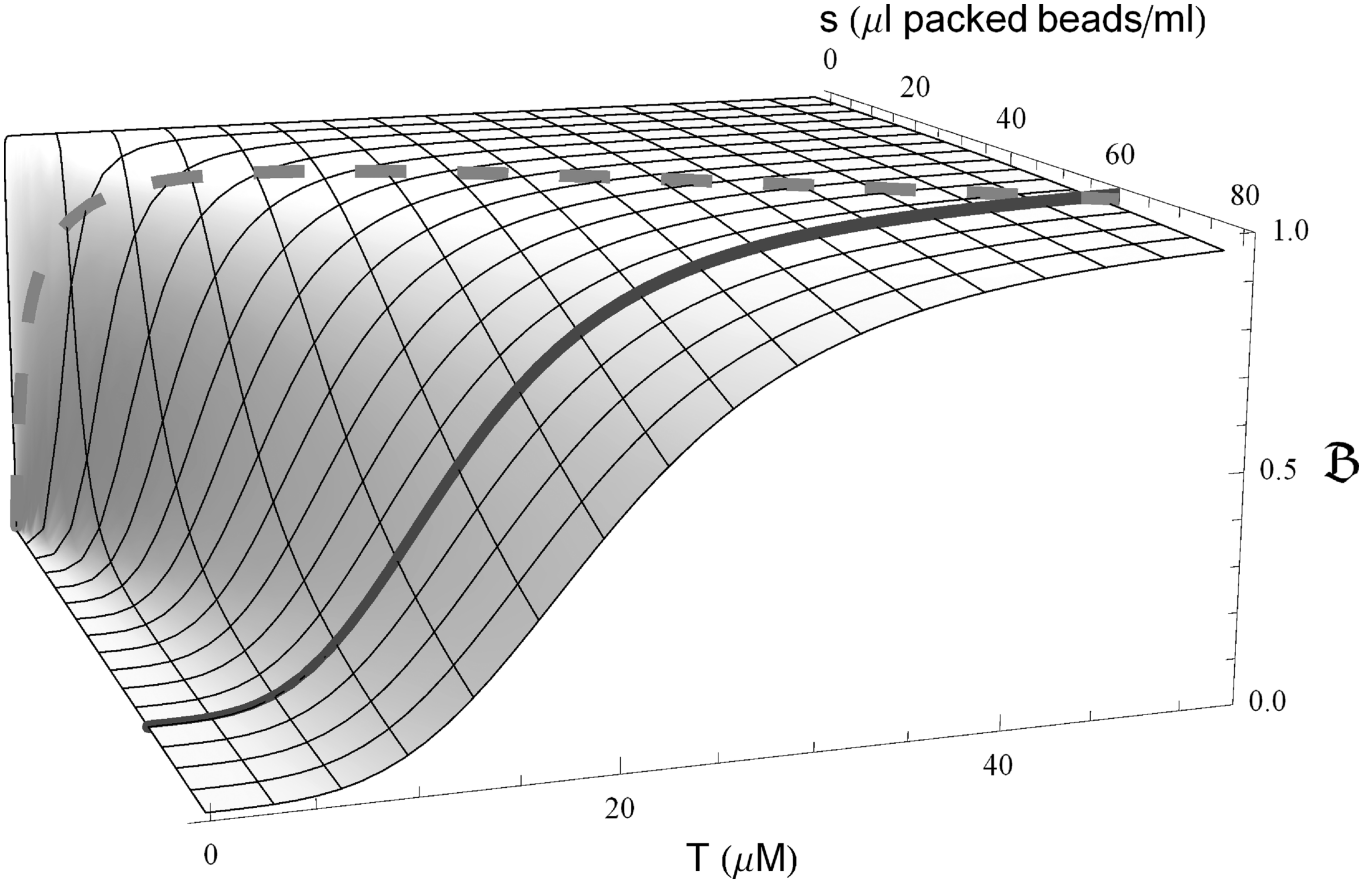
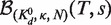
. The DDC binding function over both target (*T*) and bead (*s*) concentrations using the 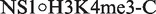 best-fit parameters is shown. The solid and dashed lines show the function for the VTSD (*s* constant) and CTSD (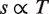) experiments, respectively.

Once the best-fit parameters have been determined (Supplementary Table S1), we can use Equation (8) to predict binding for any values of *T* and *s*. Figure 3 illustrates how the VTSD and CTSD binding curves are related to binding over the 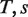-space. The contour lines at constant *T* are also of special interest: *T* will be fixed when designing a SELEX (or other affinity selection) experiment to optimize the competitive purification of ligands that bind specifically (3–8). The contour line at this value of *T* shows how the non-specific binding will vary with *s*, and thus provides a guide to choosing a value of *s* that will keep the cooperative enhancement of this undesired binding to a tolerable level.

## DISCUSSION

We measured the retention of RNA aptamer ligands by bead-immobilized histone peptide targets as target concentrations and surface densities were varied. The VTSD retention curves, for which the target-immobilizing surface area was constant, displayed inflection points and had concave log–log bound/unbound ligand ratio plots that indicated that effective affinity increased with increasing TSD. In contrast, control CTSD retention curves, for which TSD, and hence effective affinity, was high but constant, were well-fit using the Langmuir equation. The 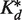
’s, the cooperative dissociation constants that govern binding at the highest TSD tested, were as much as 600 times lower than the corresponding 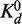
’s, the non-cooperative dissociation constants that govern binding at low TSD (e.g. NS1∘H3-C, Figure 2). We infer that cooperative binding of ligands to multiple immobilized targets can dramatically increase non-specific binding affinities.

To explain this phenomenon, we developed the ‘DDC binding model’. It is based on the recognition that, after binding the first target, a ligand can bind additional targets without additional decrease in localization entropy. Therefore, the number of targets bound, and hence the magnitude of the total free energy of binding and the effective binding affinity, increases with increasing TSD. The asymptotic DDC model is an approximation requiring only two parameters that can be used for moderate TSDs when the targets are much smaller than the ligands and the ceiling on the number of targets that can be bound by a ligand can be ignored. The complete DDC model includes a parameter 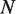, the maximum number of targets that a ligand can bind, and thereby extends accuracy to all cases. It predicts binding for any values of *T* and *s* (Figure 3) and fits the VTSD and CTSD data well for each ligand-target pair using a single set of parameters (Figure 2).

The RNA aptamers used in this study were long (120 nt), and the peptides were highly basic. These factors promote multiple strong charge–charge attractions between ligand and target and probably increased the propensity for non-specific interactions in the examples studied. Non-specific cooperation might be weaker with shorter aptamers and/or more acidic or hydrophobic targets. In this regard, the 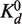
’s of the trimethylated peptide, H3K4me3-C, were higher than those of the corresponding unmodified peptide, H3-C, with all the ligands; this probably reflects decreased binding of this more hydrophobic target to the negatively charged ribonucleotides. Trimethylation had a much smaller effect on the 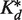
’s, which implies that the higher hydrophobicity had less effect on the subsequent bindings than on the initial binding: Although 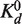 depends on the free energy of binding to the first target alone, 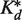 depends on the total free energy of binding, which includes contributions from subsequent binding to additional targets. We do not know the reason for the low level of cooperativity observed with the NS2∘H3-C pair.

The DDC model determined the maximum number of targets bound per ligand in all the experiments to be 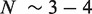. This is physically reasonable in view of the ligand, target and bead sizes: If a 120 nt aptamer were coiled to the same extent as a tRNA, it would be 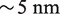 in diameter; if fully stretched across many targets, it could be up to 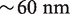 long (22). If a 25 amino acid peptide target were in an α-helical conformation, it would have a diameter of 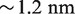 and would be 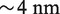 long. These are only ballpark estimates, but they illustrate that it would be possible for a ligand to bind four targets, if their surface density were high enough. This also is likely: the beads have a mean diameter of 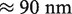 (17), and, if they were hard spheres, the targets would have a CTSD surface density of 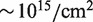, corresponding to 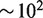
–10^4^ molecules within the area spanned by a aptamer (depending on its degree of extension). Although this is an overestimate that ignores pores and convolutions of the bead surface, it shows that it is likely that the TSD is high enough to explain the observed cooperative effects.

We assumed that the unbound targets were randomly distributed on the immobilizing surface. This is likely to be so when targets are in great excess over ligands (as in the experiments described here), but will not be so if most of the target molecules are bound. In that case, preferential binding to closely clustered targets will cause the remaining unbound targets to have lower surface density, which will reduce cooperative binding at higher ligand concentrations. This effect can be modeled mathematically, but the extent to which it modulates non-specific cooperative binding in practical applications requires further experimental study.

The classic Monod–Changeux–Wyman and Koshland–Nemethy–Filmer models of cooperative binding focus on allosteric mechanisms involving conformational transitions and/or induced fits, usually of oligomers (20). In the DDC model, cooperativity emerges as a simple consequence of the relationship between the binding free energy, localization entropy and the spatial distribution of the immobilized targets; no oligomers or conformational changes are required. Instead, the probability that a ligand will bind *n* targets is linked to the dependence of the Poisson (asymptotic model) or binomial (complete model) distribution on the TSD (see Supplementary Data sections C and D). This results in binding equations that are intermediate in complexity between the two-parameter Hill equation and the four-parameter (counting *N*) Monod–Changeux–Wyman and Koshland–Nemethy–Filmer equations. Thus, they may be useful for phenomenological cooperative binding curve fitting in other applications.

Although the experiments described here involve immobilized binding, the DCC analysis does not require immobilization: it only requires that the ligand has multiple equivalent binding sites, and that, in the absence of ligand, the targets are randomly distributed on a surface or line (see Supplementary Data section E). Therefore, the DCC model may be applicable in situations where the targets are bound or embedded, but not immobilized, in membranes or on intracellular filaments. For instance, Zhao *et al.* (23) have shown that the actin-depolymerizing factor cofilin binds 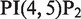 embedded in synthetic vesicles in a density-dependent, cooperative manner and the binding as a function of 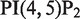 concentration in synthetic vesicles (their Figure 1A) is qualitatively similar to the VTSD binding curves shown in Figure 2. Moreover, their mutagenesis experiments suggest that the binding is driven by fairly non-specific and equivalent multiple electrostatic interactions between a single cofilin ‘ligand’ and multiple 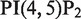 ‘targets’. Thus, the DDC model may be useful in quantitatively explaining these data. On the other hand, the model is not applicable when it is the multivalent partner that is bound to a surface, such as in the cooperative binding of ligands to multivalent receptors bound to membranes (24,25) or of proteins to DNA that is immobilized on chromatin (26,27).

The dependence of the solution-phase enrichment of a high- relative to a low-affinity ligand on *r*, *b*, *T* and the ratio of their non-cooperative dissociation constants has been extensively studied (3–8). The results presented here show that to optimize solid-phase enrichment, it will also be important to consider 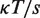. This will be especially important for efforts aimed at developing multiplexed high-throughput SELEX procedures using reaction chambers with immobilized targets. Miniaturization can be achieved using high TSDs, but this is the regime where the cooperative effects will be the largest. These can be deleterious in two ways: If cooperativity only contributes to the non-specific binding of low-affinity aptamers, it will decrease the ratio of the low- and high-affinity cooperative dissociation constants, thereby decreasing selectivity (see Supplementary Data section F). Alternatively, if the specific binding of high-affinity ligands can be augmented by cooperative non-specific binding, this may bias selection toward high-affinity ligands that bind one target specifically while also binding non-specifically to additional targets (Supplementary Data section F). This is an undesired property when it is the solution-binding properties of the ligand that are of importance.

These considerations may make it important to limit 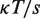. This could be achieved without changing *T*, whose optimal value is fixed by other factors (3–8), or increasing *s*, which would make the apparatus larger, by using an immobilizing substrate with a large surface area-to-volume ratio; this will reduce κ. However, increased background binding to the bare surfaces of the beads may also degrade performance, and this must also be taken into account. Once the extent of bead surface binding has been determined (e.g. by fitting the parameter *b* of Equation 2 in pilot experiments), performance can be quantitatively optimized using the function 
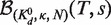
 (e.g. as exemplified by Figure 3) if the library 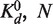, and κ and the high-affinity aptamer *K_d_*’s can be estimated. 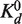 and the high-affinity *K_d_*’s are often estimated from prior experience or pilot experiments but, as far as we are aware, the experiments reported here are the first to examine *N* and κ. The range of values observed here provides some guidance, but further extensive experimental studies of cooperative binding with a wide range of targets are needed. In particular, it will be important to explore the dependence of cooperative binding on the sizes and types of ligands and targets and to determine whether cooperative non-specific binding can occur in conjunction with specific binding by high-affinity ligands.

## SUPPLEMENTARY DATA

Supplementary Data are available at NAR Online: Supplementary Table 1, Supplementary Methods and Supplementary References [21,28,29].

## FUNDING

National Institutes of Health (NIH) [DA30329 to J.T.L.]. Funding for open access charge: NIH.

*Conﬂict of interest statement.* None declared.

## Supplementary Material

Supplementary Data
