# Supplementary material for: Density-dependent cooperative non-specific binding in solid-phase SELEX affinity selection
Source: Nucleic Acids Res. 2013 May 21;41(14):7167–75. doi: 10.1093/nar/gkt477 (PMC3737557; doi:10.1093/nar/gkt477)
Supplement: Supplementary Data [file supp_41_14_7167__index.html]

Density-dependent cooperative non-specific binding in solid-phase SELEX affinity selection — Density-dependent cooperative non-specific binding in solid-phase SELEX affinity selection — Supplementary Data 

# Density-dependent cooperative non-specific binding in solid-phase SELEX affinity selection

## Supplementary Data

files

**Files in this Data Supplement:**

- Supplementary Data - pdf file
