# Supplementary material for: Density-dependent cooperative non-specific binding in solid-phase SELEX affinity selection
Source: Nucleic Acids Res. 2013 May 21;41(14):7167–75. doi: 10.1093/nar/gkt477 (PMC3737557; doi:10.1093/nar/gkt477)
Supplement: Supplementary Data [file supp_gkt477_nar-00792-f-2013-File004.pdf]

Density-dependent cooperative non-specific binding in solid-phase SELEX affinity selection
(Ozer et al.)

SUPPLEMENTARY DATA

Supplementary Theoretical Methods
Supplementary References
Supplementary Table

SUPPLEMENTARY THEORETICAL METHODS

A. NONLINEAR MODEL FITTING

This was performed using Mathematica Version 8. At the highest concentration, T and s were equal in the corresponding VTSD and CTSD experiments; therefore all the data points at this concentration were used in fitting both data sets. The logarithms of concentration variables (e.g., K_d^*) were used as parameters since this improves the accuracy of the nonlinear regression[1] (1). Because each pair of VTSD and CTSD experiments was conducted in parallel, we expected the background binding and retention constants, b and r, to be the same for both. Therefore, b, r, and $\log K_d^*$ were determined by nonlinear fitting of Eq. **2** using the Langmuir model for the CTSD data, and the same values of b and r were then used when fitting the asymptotic and complete density-dependent cooperative (DDC) models.

The measurement errors were not independent and identically distributed. Rather, since the $\mathcal{R}_i$ were measurements of the *proportions* of radioactive label that were retained, they were bounded ($0 \le \mathcal{R}_i \le 1$), so the error distributions must have been more compact for values near the bounds. To adjust for this we used weights, w_i, that were inversely proportional to the expected variance of the error. This was not known exactly, but we assumed that it had the same form as that arising when a proportion p is estimated from a collection of Bernoulli trials. In that case, the variance of p is determined from the binomial distribution to be $p(1-p)/n$, where n is the number of trials. Here we regarded n as a phenomenological parameter to be determined by the data, and so used the weight function $n/[\mathcal{R}(1-\mathcal{R})]$. However, since only relative values of the weights are important, we did not need to determine n and used $w_i = 1/[\mathcal{R}_i(1-\mathcal{R}_i)]$.

Parameter standard errors are asymptotic (i.e., were computed from the Hessian of the weighted sum of residuals). When the independent parameters $\{\phi_i\}$ had been estimated, the asymptotic standard error of another parameter φ was computed by propagation of errors using the covariance matrix Σ of the $\{\phi_i\}$ estimates as

$$\sigma_\varphi^2 = \sum_{i,j} \frac{\partial \varphi}{\partial \phi_i} \Sigma_{ij} \frac{\partial \varphi}{\partial \phi_j}\,.$$

B. REDUCED RETENTION CAUSED BY WASHING

Aptamer-target complexes may dissociate during washing. We show here that the only effect of this non-equilibirum process on the retention curves is to reduce r.

We can evaluate its effect on non-cooperative binding using the dynamic model

$$\frac{dB_{(k_+,k_-,t_b)}(T,t)}{dt} = \Theta(t_b - t) \times k_+ T[L^0 - B_{(k_+,k_-,t_b)}(T,t)] - k_- B_{(k_+,k_-,t_b)}(T,t) \quad \text{(S1)}$$

$$B_{(k_+,k_-,t_b)}(T,0) = 0\,,$$

where $B_{(k_+,k_-)}(T,t)$ is the concentration of bound ligands at time t, L^0 is the total ligand concentration, k_+ and k_- are the association and dissociation rates, t_b is the duration of the binding reaction, and $\Theta(\cdot)$ is the Heaviside step function. We assume, as was the case for this study, that the experiments are performed in great target excess so T, the unbound target concentration, is essentially constant. Also, because the wash volumes were large, rebinding during the wash period is ignored.

Solving Eq. **S1** for $t > t_b$ determines the solution binding at time $t = t_b + t_w$, where t_w is the total wash duration, to be

$$\mathcal{B}_{(k_+,k_-,t_b)}(T, t_b + t_w) = \frac{B_{(k_+,k_-,t_b)}(T, t_b + t_w)}{L^0} = (1 - e^{-(k_- + k_+ T)t_b}) e^{-k_- t_w} \mathcal{B}_{K_d}(T)\,, \quad \text{(S2)}$$

where $K_d = k_-/k_+$ and $\mathcal{B}_{K_d}(T)$ is the equilibrium binding fraction given by Eq. **1**. As long as binding phase has progressed to equilibrium [i.e., $t_b \gg (k_- + k_+ T)^{-1}$], the only non-equilibrium effect will be that of washing, and this will simply introduce a T-independent factor $e^{-k_- t_w} < 1$ that will reduce r.[2]

To analyze solid-phase binding we refer to Eq. **5** and assume, as seems likely, that the time-scale of the $U \rightleftharpoons B_1$ transition is much slower than that of the subsequent $B_n \rightleftharpoons B_{n+1}$ transitions. Hence, we can use an adiabatic approximation wherein the different bound states are assumed to be in equilibrium with each other so that $B(t) \equiv \sum_n B_n(t) \propto B_1(t)$. Therefore, Eq. **S2** is still applicable with the replacement of k_+ and k_- by the TSD-dependent cooperative rate constants. Thus, in this case as well, washing only reduces r and does not change the shape of the binding curve.

[1] Nonlinear regression is equivalent to linear regression using linear approximations to the model function at the data points. Accuracy is improved by using parameters that improve the linear approximation, i.e., parameters that reduce the contributions from the second derivatives. When K_d is used, this contribution is $[(\Delta K_d)^2/2]\,|d^2\mathcal{B}_{K_d}/dK_d^2| = (\Delta K_d)^2/(K_d + T)^3$. When $\log K_d$ is used, this is $[(\Delta \log K_d)^2/2]\,|d^2\mathcal{B}_{K_d}/d(\log K_d)^2| = (\Delta K_d)^2(1 - T/K_d)/[2(K_d + T)^3]$. The ratio of the $\log K_d$ to the K_d second derivative contribution is $|1 - T/K_d|/2$, which vanishes at $T = K_d$ and is less than one over most of the relevant range of concentrations.

[2] Non-equilibrium washing effects will affect the relative binding of different aptamers with different dissociation rates, but this is not relevant here.

C. ASYMPTOTIC DDC MODEL

To set the stage we first analyze non-cooperative solution binding using partition functions in a manner that generalizes to the cooperative immobilized case.

headingSolution binding We assume that a ligand and a target must be located within the same "binding volume" v to bind, that the total volume V_0 equals the "reference volume" (1 liter for molar concentration units), and that the concentration of unbound targets is T. Since the ligands do not interact, we can consider one individually. It can be located in one of the

$$\Omega_V^L = V_0/v \tag{S3}$$

binding volumes, which is the number of its (discrete) spatial configuration states. Including the correct Boltzmann counting factor, the targets can be in

$$\Omega_V^T = \frac{(V_0/v)^{TN_aV_0}}{(TN_aV_0)!}$$

possible configuration states, where N_a is Avogadro's number. Since the complete configuration space of unbound states is the outer product of the ligand and target spaces and there is an unbound microstate corresponding to each configuration, there are $\Omega_V^L \times \Omega_V^T$ unbound microstates. Without loss of generality we can assign each of them zero free-energy, so the partition function of the unbound states is

$$Z^U = \Omega_V^L \Omega_V^T \,.$$

The partition function of the bound states also factors into a product of ligand- and target-dependent terms, but the latter is now more complicated. As before, the ligand can be in any one of the Ω_V^L binding volumes. However, only those target configurations having at least $m \geq 1$ targets in the binding volume containing the ligand can contribute to the Boltzmann sum over the bound microstates, and the contribution will depend on m. Therefore, we begin by counting the number of ways of having m randomly distributed targets in this binding volume. It is easy to do this by working "backwards" from the corresponding probability: This is the Poisson distribution $\mathcal{P}(m;\mu_V)$,

$$\mathcal{P}(m;\mu_V) = \frac{\mu_V^m e^{-\mu_V}}{m!} \,,$$

where $\mu_V = TN_av$ is the mean number of targets per binding volume.[3] Since this probability is the ratio between the number of target configurations satisfying the criterion, $(\Omega_V^T)_m$, and the total number of target configurations, Ω_V^T,

$$(\Omega_V^T)_m = \Omega_V^T \mathcal{P}(m;\mu_V) \,.$$

Thus, the number of configuration states having m targets colocalized with the ligand is $\Omega_V^L \times (\Omega_V^T)_m$. For each such configuration state, there are

$$\omega_{m1}^B = \binom{m}{1} = m$$

ways of selecting one of the colocalized targets for binding to the ligand. Therefore, there are $\Omega_V^L \times (\Omega_V^T)_m \times \omega_m^B$ bound microstates having m colocalized targets. Summing over m, the total number of bound microstates is

$$\begin{aligned}\Omega^B &= \Omega_V^L \sum_{m=1}^{\infty} (\Omega_V^T)_m \omega_{m1}^B \\ &= \Omega_V^L \Omega_V^T \sum_{m=1}^{\infty} m\mathcal{P}(m;\mu_V) \\ &= \Omega_V^L \Omega_V^T \mu_V \,.\end{aligned}$$

All of the bound microstates have the same "intrinsic" binding free-energy for a colocalized ligand and target, $\Delta G'$.[4] Therefore, denoting temperature as τ, Boltzmann's constant as k_B, and $\beta_B \equiv (k_B\tau)^{-1}$, the bound state partition function is

$$\begin{aligned}Z^B &= \Omega^B e^{-\beta_B \Delta G'} \\ &= T(N_aV_0)\frac{v}{V_0} Z^U e^{-\beta_B \Delta G'}\end{aligned} \tag{S4}$$

and the bound/unbound ligand ratio is

$$\frac{Z^B}{Z^U} = \rho_{K_d}(T) = \frac{T}{K_d} \tag{S5}$$

where
$$\begin{aligned}K_d &= (N_aV_0)^{-1} e^{\beta_B \Delta G} = (N_av)^{-1} e^{\beta_B \Delta G'} \\ \Delta G &= \Delta G' - \tau \Delta S^v \\ \Delta S^v &= k_B \log\left(\frac{v}{V_0}\right) \,,\end{aligned}$$

and ΔS^v is the entropy of localization, which corresponds to the v/V_0 prefactor in Eq. **S4**. This proves Eq. **4** and, in combination with Eq. **3**, proves Eq. **1**.

[3] To be precise, the probability is the binomial distribution for m successes in N trials with success probability p. However, since TN_aV_0 is huge, we can use the Poisson approximation.

[4] We use the notation of Ref. 1, which discusses the separation of binding free-energy into a spatial component, the "connection Gibbs energy", and the intrinsic component.

Immobilized binding

Denoting the bead[5] concentration (expressed as the fraction of the reaction volume occupied by packed beads) as s and the number of moles of binding volumes on the surface of a liter of packed beads as C_s, the ligand has Ω_V^L possible configuration states in solution and

$$\Omega_\sigma^L = sC_s N_a V_0 \ll \Omega_V^L \tag{S6}$$

states on the bead surface. The targets are immobilized to the bead surface in one of

$$\Omega_\sigma^T = \frac{(sC_s N_a V_0)^{TN_a V_0}}{(TN_a V_0)!} \tag{S7}$$

possible configuration states. Therefore, ignoring (vanishingly small) terms of $O(\Omega_\sigma^L/\Omega_V^L)$, the partition function of the unbound states is

$$Z^U = \Omega_V^L \Omega_\sigma^T . \tag{S8}$$

We calculate the number of ways of having m randomly distributed targets located within a specific binding volume on the bead surface by again working backwards from the probability of this event assuming random placement of the targets on the bead surface. This is $\mathcal{P}(m;\mu_\sigma)$, where

$$\mu_\sigma = T/(sC_s) \tag{S9}$$

is the mean number of targets per binding volume on the bead surface. Therefore, the number of ways of having m randomly distributed targets in a specified binding volume is

$$(\Omega_\sigma^T)_m = \Omega_\sigma^T \mathcal{P}(m;\mu_\sigma) .$$

and the number of configuration states having m targets colocalized with the ligand is $\Omega_\sigma^L \times (\Omega_\sigma^T)_m$. For each of these configuration states, there are

$$\omega_{mn}^B = \binom{m}{n} = \frac{m!}{(m-n)!n!} \tag{S10}$$

ways of selecting n of the colocalized targets for binding. Thus, there are $\Omega_\sigma^L \times (\Omega_\sigma^T)_m \times \omega_{mn}^B$ microstates having m ligand-colocalized targets and n ligand-bound targets. Summing over m, $n \leq m$, the total number of microstates having n targets bound to a ligand is

$$\begin{aligned}\Omega_n^B &= \Omega_\sigma^L \Omega_\sigma^T \sum_{m=n}^{\infty} \mathcal{P}(m;\mu_\sigma) \binom{m}{n} \\ &= \Omega_\sigma^L \Omega_\sigma^T \frac{\mu_\sigma^n}{n!} .\end{aligned}$$

Each of these bound microstates has residual binding free energy $\Delta G_1' + (n-1)\Delta G_+'$, where $\Delta G_1'$ and $\Delta G_+'$ are the residual free-energies for binding the first and subsequent colocalized targets, respectively. Therefore, the partition function for states having $n \geq 1$ bound targets is

$$\begin{aligned}Z_n^B &= \Omega_n^B e^{-\beta_B[\Delta G_1' + (n-1)\Delta G_+']} \\ &= \Omega_\sigma^L \Omega_\sigma^T e^{-\beta_B(\Delta G_1' - \Delta G_+')} e^{\mu_\sigma e^{-\beta_B \Delta G_+'}} \mathcal{P}(n;\mu_\sigma e^{-\beta_B \Delta G_+'}) .\end{aligned}$$

Summing over n, $1 \leq n$, gives the complete bound state partition function

$$\begin{aligned}Z^B &= \sum_{n=1}^{\infty} Z_n^B \\ &= \Omega_\sigma^L \Omega_\sigma^T e^{-\beta_B(\Delta G_1' - \Delta G_+')} e^{\mu_\sigma e^{-\beta_B \Delta G_+'}} \sum_{n=1}^{\infty} \mathcal{P}(n;\mu_\sigma e^{-\beta_B \Delta G_+'}) \\ &= sC_s N_a v Z^U e^{-\beta_B(\Delta G_1' - \Delta G_+')} \left(e^{T/(sC_s) e^{-\beta_B \Delta G_+'}} - 1 \right),\end{aligned} \tag{S11}$$

where we used Eqs. **S3**, **S6**, **S8**, and **S9** and the Poisson distribution's summation property to get the last line. Therefore,

$$\frac{Z^B}{Z^U} = \rho_{(K_d^0,\bar{\kappa})}(T) = \frac{s}{\bar{\kappa} K_d^0} \left(e^{\bar{\kappa} T/s} - 1 \right),$$

where

$$\begin{aligned}K_d^0 &= (N_a v)^{-1} e^{\beta_B \Delta G_1'} = (N_a V_0)^{-1} e^{\beta_B \Delta G_1} \\ \Delta G_1 &= \Delta G_1' - \tau \Delta S^v \\ \Delta S^v &= k_B \log\left(\frac{v}{V_0}\right) \\ \bar{\kappa} &= \frac{1}{C_s} e^{-\beta_B \Delta G_+'} ,\end{aligned}$$

thereby proving Eq. **6**.

Comparing this with Eq. **S5**, we see that K_d^0 would be the same as the solution binding dissociation constant if $\Delta G_1 = \Delta G$, i.e., if a ligand were to bind colocalized solvated and immobilized targets with the same affinity. $\bar{\kappa}$, the asymptotic cooperativity constant, is determined by $\Delta G_+'$. Z_n^B is proportional to a Poisson distribution with expectation value $\bar{\kappa}T/s$ and the probabilities of binding one or two targets are the same when $\bar{\kappa}T/s = 2$. Thus, $\bar{\kappa}$ determines the scale of TSDs at which cooperativity becomes significant.

Relationship to intrinsic binding energy

The decomposition of the binding free-energy in terms of $\Delta G_1'$, $\Delta G_+'$, and the translational entropy component $T\Delta S^v$ is similar in principle to Jencks' (2) decomposition of binding free-energy into spatial (translational plus rotational) entropy, strain, and the intrinsic components. However, the decomposition used here, which is optimized for the analysis of the sequential binding process of Eq. **5**, differs in detail. While $\Delta G_1'$ excludes the change in translational entropy, it includes the change in rotational entropy and $\Delta G_+'$ includes

[5] The analysis generalizes to any immobilizing substrate.

any contribution from strain that may be present. However, we expect strain contributions to be small, so that $\Delta G'_+$ is approximately equal to the intrinsic binding free-energy for non-specific binding of the ligand once the first binding has induced any global conformational changes that are needed for non-specific binding.

Number of bound targets

The mean number of targets bound by a ligand conditional on the ligand being bound is the Boltzmann-weighted average of n over the bound states. Noting that $-\partial Z_n^B/\partial(\beta_B \Delta G'_+) = (n-1)Z_n^B$ and $\partial/\partial(\beta_B \Delta G'_+) = -\partial/\partial \log\bar{\kappa}$ and using Eq. **S11** we get

$$\begin{aligned}\bar{n}_{(K_d^0,\bar{\kappa})}(T,s) &= (Z^B)^{-1}\sum_{n=1}^{\infty} n Z_n^B \\ &= (Z^B)^{-1}\sum_{n=1}^{\infty}\left(-\frac{\partial}{\partial \beta_B \Delta G'_+}+1\right) Z_n^B \\ &= \frac{\partial \log Z^B}{\partial \log \bar{\kappa}}+1 \\ &= \frac{\bar{\kappa}T/s}{1-e^{-\bar{\kappa}T/s}}\,.\end{aligned}$$

Reparameterization for VTSD experiments

In a VTSD experiment $s = s^*$, and it is convenient to define

$$\bar{\gamma} \equiv \frac{\bar{\kappa}K_d^0}{s^*}$$

and to rewrite Eq. **6** as

$$\rho^{\mathsf{VTSD}}_{(K_d^0,\bar{\gamma})}(T) = (e^{\bar{\gamma}T/K_d^0}-1)/\bar{\gamma}\,,$$

where the dependence of $\bar{\gamma}$ on s^* is implicit. While cooperativity will increase binding whenever $\bar{\gamma} > 0$, the retention curve will have an inflection point only when $\bar{\gamma} > 2$. This corresponds to $\bar{\kappa}K_d^0/s^* > 2$, which means that there will be an inflection point in a VTSD experiment when the probability of binding one or two targets is the same when $T = K_d^0$. The inflection point will be at $T_{\mathsf{infl}} = (K_d^0/\bar{\gamma})\log(\bar{\gamma}-1)$.

It is sometimes useful to express Eq. **6** in terms of $K_{0.5}(K_d^0,\bar{\gamma})$, the half-binding *dissociation concentration* at which

$$\begin{aligned}\rho_{(K_d^0,\bar{\gamma})}(T)\Big|_{T=K_{0.5}(K_d^0,\bar{\gamma})} &= 1 \\ \mathcal{B}_{(K_d^0,\bar{\gamma})}(T,s)\Big|_{T=K_{0.5}(K_d^0,\bar{\gamma})} &= 1/2\end{aligned}$$

and $\bar{n}(\kappa)$, the mean number of targets bound to a bound ligand when $T = K_{0.5}(K_d^0,\bar{\gamma})$:

$$\begin{aligned}K_{0.5}(K_d^0,\bar{\gamma}) &= \frac{K_d^0}{\bar{\gamma}}\log(1+\bar{\gamma}) \\ \bar{n}(\bar{\gamma}) &= \frac{(1+\bar{\gamma})\log(1+\bar{\gamma})}{\bar{\gamma}}\,.\end{aligned}$$

$\bar{n}(\gamma)$ and $K_{0.5}(K_d^0,\bar{\gamma})$ are often determined more accurately by VTSD data than K_d^0 and $\bar{\gamma}$ because the first two parameters primarily depend on the (often most accurate) data in the mid-range of binding. The asymptotic DDC equation can reparameterized in terms of these variables using the inverse relations

$$\begin{aligned}K_d^0(K_{0.5},\bar{n}) &= -K_{0.5}/W(-\bar{n}e^{-\bar{n}}) \\ \bar{\gamma}(\bar{n}) &= -1-\bar{n}/W(-\bar{n}e^{-\bar{n}})\,,\end{aligned}$$

where $W(x)$ is the principal branch of the Lambert W function.[6]

D. COMPLETE DDC MODEL

In the complete model we assume that the ligand has N non-overlapping, non-specific binding sites, where N is determined by the size of the target in relation to the ligand and possibly other factors. (While this is a simplification—the positions of potential non-specific binding may not be fixed—we expect that the error it introduces will be small.) The analysis in this case follows that of the asymptotic case. Ω_σ^L (Eq. **S6**) and Ω_σ^T (Eq. **S7**) are the same as in the asymptotic case, but $(\Omega_\sigma^T)_m$ and its dependent quantities are different. We calculate the number of ways that m targets can be colocalized with these binding sites by again working backwards from the probability of the event. Since there are μ_σ targets per binding volume and N binding sites per binding volume, the number of targets per binding site is μ_σ/N and the probability of having m targets colocalized with the ligand binding sites is $\mathrm{Bin}(m;\mu_\sigma/N,N)$, where

$$\mathrm{Bin}(m;p,N) = \binom{N}{m} p^m (1-p)^{N-m}$$

is the binomial distribution. Therefore, the number of target configurations having m targets colocalized with binding sites of a ligand at a specific location is

$$(\Omega_\sigma^T)_m = \Omega_\sigma^T\,\mathrm{Bin}(m;\mu_\sigma/N,N)\,.$$

[6]We can also parameterize the binding curves using K_d^0 and $K_{0.5}$ by inverting these equations to get $\bar{\gamma} = -1-(K_d^0/K_{0.5})W_{-1}[-(K_{0.5}/K_d^0)\exp(-K_{0.5}/K_d^0)]$, where $W_{-1}(x)$ is the lower of the two real branches of the Lambert W-function over $-\exp(-1) < x < 0$.

ω_{mn}^B (Eq. **S10**) is unchanged from the asymptotic analysis. Multiplying Ω_σ^L, $(\Omega_\sigma^T)_m$, and ω_{mn}^B and summing over m, $n \le m \le N$, gives

$$\begin{aligned}\Omega_n^B &= \Omega_\sigma^L \Omega_\sigma^T \sum_{m=n}^{N} \mathrm{Bin}(m;\mu_\sigma/N,N)\binom{m}{n}\\ &= \Omega_\sigma^L \Omega_\sigma^T (1-\mu_\sigma/N)^N \frac{N!}{n!}\sum_{m=n}^{N}\frac{[\mu_\sigma/(N-\mu_\sigma)]^m}{(N-m)!(m-n)!}\\ &= \Omega_\sigma^L \Omega_\sigma^T \binom{N}{n}\left(\frac{\mu_\sigma}{N}\right)^n\end{aligned}$$

Multiplying this by the Boltzmann factor gives the partition function for states having n bound targets

$$\begin{aligned}Z_n^B &= \Omega_n^B e^{-\beta_B[\Delta G_1' + (n-1)\Delta G_+']}\\ &= \Omega_\sigma^L \Omega_\sigma^T e^{-\beta_B(\Delta G_1' - \Delta G_+')}\left(1+\frac{\mu_\sigma}{N}e^{-\beta_B \Delta G_+'}\right)^N \times\\ &\quad \mathrm{Bin}\left(n;\frac{(\mu_\sigma/N)e^{-\beta_B\Delta G_+'}}{1+(\mu_\sigma/N)e^{-\beta_B\Delta G_+'}},N\right) \qquad \text{(S12)}\end{aligned}$$

Summing over n, $1 \le n \le N$, and using the binomial distribution's summation property gives

$$\begin{aligned}Z^B &= \sum_{n=1}^{N} Z_n^B\\ &= \Omega_\sigma^L \Omega_\sigma^T e^{-\beta_B(\Delta G_1' - \Delta G_+')}\left(1+\frac{\mu_\sigma}{N}e^{-\beta_B \Delta G_+'}\right)^N \times\\ &\quad \sum_{n=1}^{N}\mathrm{Bin}\left(n;\frac{(\mu_\sigma/N)e^{-\beta_B\Delta G_+'}}{1+(\mu_\sigma/N)e^{-\beta_B\Delta G_+'}},N\right)\\ &= \Omega_\sigma^L \Omega_\sigma^T e^{-\beta_B(\Delta G_1' - \Delta G_+')}\left[(1+\frac{\mu_\sigma}{N}e^{-\beta_B \Delta G_+'})^N - 1\right].\end{aligned}$$

Using Eqs. **S3**, **S6**, and **S8** and the definition of K_d^0 we get

$$\begin{aligned}\frac{Z^B}{Z^U} &= \rho_{(K_d^0,\kappa,N)}(T,s)\\ &= \frac{(N-1)s}{N\kappa K_d^0}\left\{\left[1+\frac{\kappa T}{s(N-1)}\right]^N - 1\right\}, \qquad \text{(S13)}\end{aligned}$$

where $\qquad \kappa \equiv \dfrac{N-1}{NC_s}e^{-\beta_B\Delta G_+'}$,

thereby proving Eq. **8**. In this case, Z_n^B is proportional to a binomial distribution with expectation value $N\kappa(T/s)/(N-1+\kappa T/s)$. The probabilities of binding one or two targets are the same when $\kappa T/s = 2$.

As before, we calculate the number of bound targets using $\bar{n}_{(K_d^0,\kappa,N)} = \partial \log Z^B/\partial\log\kappa + 1$. This gives

$$\bar{n}_{(K_d^0,\kappa,N)}(T,s) = N\frac{\kappa T/[s(N-1)])}{\{(1+\kappa T/[s(N-1)]\}(1-\{1+\kappa T/[s(N-1)]\}^{-N})},$$

which, as expected, has the limits

$$\lim_{T/s\to 0}\bar{n}_{(K_d^0,\kappa,N)}(T,s) = 1$$
$$\lim_{T/s\to\infty}\bar{n}_{(K_d^0,\kappa,N)}(T,s) = N.$$

For analyzing VTSD data alone, it is convenient to define

$$\gamma \equiv \frac{N\kappa K_d^0}{s(N-1)}$$

and to rewrite Eq. **S13** as

$$\rho_{(K_d^0,\gamma,N)}(T) = \left\{[1+(\gamma/N)(T/K_d^0)]^N - 1\right\}\Big/\gamma.$$

This has the same non-cooperative behavior for small $\kappa T/s$ as the asymptotic equation,

$$\rho_{(K_d^0,\gamma,N)}(T) \sim \frac{T}{K_d^0} \qquad (\text{as } \kappa T/s \to 0), \qquad \text{(S14)}$$

but it has different behavior for large $\kappa T/s$,

$$\rho_{(K_d^0,\gamma,N)}(T) = \gamma^{N-1}[(T/K_d^0)/N]^N \quad (\text{as } \kappa T/s \to \infty), \qquad \text{(S15)}$$

which corresponds to the concurrent binding of N targets. The dissociation concentration is

$$K_{0.5}(K_d^0,\gamma,N) = \frac{NK_d^0}{\gamma}\left[(1+\gamma)^{1/N} - 1\right]$$

and the mean number of targets bound when $T = K_{0.5}(K_d^0,\gamma,N)$ is

$$\bar{n}(\gamma,N) = \frac{N(1+\gamma)}{\gamma}\left[1-(1+\gamma)^{-1/N}\right].$$

It is easily verified that all these equations go to their asymptotic counterparts as $N \to \infty$.

E. APPLICATION OF THE DDC BINDING MODEL TO MOBILE TARGETS

Although the DDC binding equations were derived assuming that the targets are immobilized, the same analysis applies when they are not. They only require that the targets be restricted to a two-dimensional (or even one-dimensional) subspace. This follows because target mobility does not affect the counting of the target conformational states, so the Boltzmann sums are not affected: in both cases the sum over bound states contains the term Ω_n^B that includes the same Poisson (in the asymptotic case) or binomial (in the complete case) distribution, which represents the entropy associated with target localization. However, the physical interpretations are different: in immobilized binding this term comes from the probability that the ligand is located in a region having the specified number of targets; in binding to mobile targets that can move within the lower-dimensional subspace it comes from the entropy change occurring when the specified number of targets are moved into the binding volume of an arbitrarily placed ligand. The key factor in both cases is that the large reduction in entropy that occurs when the ligand is localized from the three-dimensional solution to the lower-dimensional subspace only occurs for the first, but not the subsequent, bindings.

F. COMBINED SPECIFIC AND COOPERATIVE NON-SPECIFIC BINDING

Here we consider the extent to which cooperative non-specific binding will affect the relative affinities of a low-affinity ligand that binds only non-specifically and a high-affinity ligand that possesses a specific binding site. This will depend on whether the conformational restrictions imposed by specific binding preclude or permit additional non-specific binding. If, as seems likely, it were precluded, then the dissociation constant of the high-affinity ligand will be unchanged but that of the low-affinity ligand would be decreased from K_d^0 to K_d^*. Changes as large as those observed in the experiments reported here would decrease enrichment by two orders-of-magnitude. At the other extreme, if specific binding did not diminish additional non-specific cooperative binding, we show below that the DDC equation predicts that the effective affinity of the high-affinity ligand would be increased even more than that of the low-affinity ligand; this would cause up to an N–fold *increase* in the inverse ratio of the cooperative dissociation constants. Thus, in this case as well, cooperative binding will distort the observed (effective) affinity. This will be particularly problematic in a SELEX experiment if the cooperative effect differs between different specifically binding aptamers: because of this, the ranking of effective affinities, which determines which aptamers will be selected, will not match the ranking of the non-cooperative affinities, which determines the utility of the aptamers in an application.

We analyze the change in effective affinity occurring in the second case by modifying the procedure used in Sec. D. The calculation of Ω_n^B is unchanged. However, to calculate $Z_n^{B\star}$, the analog of Z_n^B, we must treat the configurations that include or do not include the specific binding site separately and account for the difference between the specific and non-specific binding free-energies. Since there are $\binom{N-1}{n-1}$ ways of choosing the specific binding site and $n-1$ non-specific binding sites, $\binom{N-1}{n}$ ways of choosing all n target-bound sites from the $N-1$ non-specific binding sites, and a total of $\binom{N}{n}=\binom{N-1}{n-1}+\binom{N-1}{n}$ ways of choosing n bound sites from the N potential binding sites on the ligand, the fraction of the Ω_n^B configurations that include the specific binding site is $\binom{N-1}{n-1}/\binom{N}{n}=n/N$, and the fraction that does not is $1-n/N$. Combining this with the appropriate Boltzmann factors gives

$$\begin{aligned} Z_n^{B\star} &= \Omega_n^B \left[\frac{n}{N} e^{-\beta_B \Delta G'_\star} + \left(1-\frac{n}{N}\right) e^{-\beta_B \Delta G'_1}\right] e^{-(n-1)\beta_B \Delta G'_+} \\ &= \left[\frac{n}{N}\left(e^{-\beta_B(\Delta G'_\star - \Delta G'_1)}-1\right)+1\right] Z_n^B \,, \end{aligned}$$

where $\Delta G'_\star$ is the residual free-energy for specific binding to a target located in the binding region and Z_n^B is given by Eq. **S12**. In the first line, the first term represents states having targets bound to the specific site plus $n-1$ non-specific sites and the second term represents states having targets bound to n non-specific sites.

Summing over n, $1 \leq n \leq N$, evaluating the first term using the formula for the mean of the binomial distribution and the second term as before, we get the high-affinity ligand bound-state partition function

$$\begin{aligned} Z^{B\star} &= \Omega_\sigma^L \Omega_\sigma^T \left(e^{-\beta_B \Delta G'_\star} - e^{-\beta_B \Delta G'_1}\right) \times \\ &\quad \frac{\mu_\sigma}{N}\left(1+\frac{\mu_\sigma}{N} e^{-\beta_B \Delta G'_+}\right)^{N-1} + Z^B \end{aligned}$$

Dividing by Z^U gives

$$\begin{aligned} \frac{Z^{B\star}}{Z^U} &= \rho^\star_{(K_d^\star, K_d^0, \kappa, N)}(T,s) \\ &= T\left(\frac{1}{K_d^\star} - \frac{1}{K_d^0}\right)\left(1+\frac{\kappa T}{s(N-1)}\right)^{N-1} + \rho_{(K_d^0,\kappa,N)}(T,s) \,, \end{aligned}$$

where K_d^0 and κ are defined as before and

$$K_d^\star = \frac{N}{N_a v}\left[e^{-\beta_B \Delta G'_\star} + (N-1) e^{-\beta_B \Delta G'_1}\right]^{-1} \tag{S16}$$

is the dissociation constant in the absence of cooperative binding.[7]

[7] The factor of N in the numerator of the prefactor in Eq. **S16** arises because v is the total binding volume of all N binding sites. The factor $N-1$ arises because this is the number of non-specific binding sites. Any difference between the binding volumes of the specific and non-specific sites is included in $\Delta G'_\star$.

$\rho^{\star}_{(K^{\star}_d, K^0_d, \kappa, N)}(T,s)$ has the limiting and asymptotic properties

$$\lim_{K^{\star}_d \to K^0_d} \rho^{\star}_{(K^{\star}_d, K^0_d, \kappa, N)}(T,s) = \rho_{(K^0_d, \kappa, N)}(T,s)$$

$$\lim_{N \to 1} \rho^{\star}_{(K^{\star}_d, K^0_d, \kappa, N)}(T,s) = \frac{T}{K^{\star}_d}$$

$$\rho^{\star}_{(K^{\star}_d, K^0_d, \kappa, N, K^{\star})}(T,s) \sim \frac{T}{K^{\star}_d} \qquad \text{(as } \kappa T/s \to 0\text{)}$$

$$\rho^{\star}_{(K^{\star}_d, K^0_d, \kappa, N)}(T,s) \sim T^N \left(\frac{1}{K^{\star}_d} - \frac{N-1}{N K^0_d} \right) \left(\frac{\kappa}{s(N-1)} \right)^{N-1} \qquad \text{(as } \kappa T/s \to \infty\text{)}\,.$$

The first limit corresponds to the conversion of a high-affinity ligand to a non-specifically binding ligand as the affinity of the specific binding site is reduced to that of the non-specific sites; i.e., as $K^{\star}_d \to K^0_d$.[8] The $N \to 1$ limit corresponds to Langmuir binding of a high-affinity ligand when there are no additional non-specific binding sites. The asymptotic behavior as $\kappa T/s \to 0$ corresponds to non-cooperative binding of a single target to either the single specific site or one of the $N-1$ non-specific sites. The asymptotic behavior as $\kappa T/s \to \infty$ corresponds to simultaneous binding to the specific site and all the $N-1$ non-specific sites.

Comparing the asymptotic behaviors with Eqs. **S14** and **S15**,

$$\lim_{\kappa T/s \to 0} \frac{\rho^{\star}_{(K^{\star}_d, K^0_d, \kappa, N)}(T,s)}{\rho_{(K^0_d, \kappa, N)}(T,s)} = \frac{K^0_d}{K^{\star}_d} \qquad \text{(non-cooperative)}$$

$$\lim_{\kappa T/s \to \infty} \frac{\rho^{\star}_{(K^{\star}_d, K^0_d, \kappa, N)}(T,s)}{\rho_{(K^0_d, \kappa, N)}(T,s)} = \frac{N K^0_d}{K^{\star}_d} - (N-1) \qquad \text{(highly cooperative)}.$$

This shows that cooperation, which occurs when $\kappa T/s \gtrsim 1$, can increase the ratio between the affinities of the specific and non-specific ligands by up to N-fold relative to their non-cooperative ratio, which occurs when $\kappa T/s \ll 1$. This is because the combinatoric advantage of the multiple non-specific binding sites is removed when all the binding sites are occupied.

SUPPLEMENTARY REFERENCES

1. Johnson, M. L. (2008) Nonlinear least-squares fitting methods. *Meth. Cell. Biol.* **84**, 781–805.
2. Jencks, W. P. (1981) On the attribtution and additivity of binding energies. *Proc Natl Acad Sci USA* **78**, 4046–4050.
3. Limpert, E. and Stahel, W. A. (2011) Problems with using the normal distribution - and ways to improve quality and efficiency of data analysis. *PLOS One* **6**, e21403.

[8]This corresponds to $\Delta G'_{\star} \to \Delta G'_1$.

SUPPLEMENTARY TABLE

Table S1. DDC and Langmuir best-fit parameters to the retention data of Fig. 2.

	DDC model (VTSD and CTSD)				Langmuir (CTSD)
	$K_d^0\,(\mu M)$	N	$\kappa\,(\mathrm{mM}^{-1})$	$K_d^*\,(\mu M)$	$K_d^*\,(\mu M)$
NS1∘H3-C	$61 \overset{\times}{\div} 1.9$	2.9 ± 0.3	$72 \overset{\times}{\div} 1.8$	$0.25 \overset{\times}{\div} 1.2$	$0.21 \overset{\times}{\div} 1.3$
NS1∘H3K4me3-C	$460 \overset{\times}{\div} 2.6$	3.6 ± 0.4	$57 \overset{\times}{\div} 1.9$	$0.93 \overset{\times}{\div} 1.1$	$0.76 \overset{\times}{\div} 1.2$
NS2∘H3-C	$9.7 \overset{\times}{\div} 1.2$	nd	$4.6 \overset{\times}{\div} 1.8$	$1.0 \overset{\times}{\div} 1.2$	$0.80 \overset{\times}{\div} 1.1$
NS2∘H3K4me3-C	$48 \overset{\times}{\div} 1.5$	3.6 ± 0.9	$19 \overset{\times}{\div} 1.7$	$1.1 \overset{\times}{\div} 1.1$	$0.88 \overset{\times}{\div} 1.2$
N70∘H3-C	$59 \overset{\times}{\div} 2.0$	nd	$15 \overset{\times}{\div} 2.5$	$1.6 \overset{\times}{\div} 1.2$	$1.6 \overset{\times}{\div} 1.3$
N70∘H3K4me3-C	$140 \overset{\times}{\div} 2.4$	nd	$18 \overset{\times}{\div} 2.2$	$1.2 \overset{\times}{\div} 1.2$	$1.2 \overset{\times}{\div} 1.3$

Arithmetic standard errors are displayed for N, and geometric standard errors (denoted by $\overset{\times}{\div}$) are displayed for all dissociation constants. [The geometric standard error of K is the exponential of the arithmetic standard error of $\log K$ (3), the parameter used in the nonlinear regression (see Sec. A.)] nd: not determined accurately—in these cases the standard error is greater than half the estimated value.
